# Adapting the social practice of beekeeping to a changing climate

**DOI:** 10.1007/s13280-026-02354-6

**Published:** 2026-02-23

**Authors:** Simon P. Meisch, Scott R. Bremer, Etienne Dunn-Sigouin, Manuel Hempel

**Affiliations:** 1 International Centre for Ethics in the Sciences and Humanities, University of Tübingen, Wilhelmstr. 56, 72074 Tübingen, Germany; 2 Centre for the Study of the Sciences and Humanities, University of Bergen, Parkveien 9, 5007 Bergen, Norway; 3 NORCE Norwegian Research Centre, Bjerknes Centre for Climate Research, Jahnebakken 5, 5007 Bergen, Norway; 4 University of Bergen, Bergen, Norway

**Keywords:** Beekeeping, Climate adaptation, Micro-adaptation, Social practices, Temporality, more-than-human interactions

## Abstract

Beekeeping offers a lens to study climate change, linking ecological dynamics with cultural practices, technologies, and livelihoods. While ecological impacts on bees and pollination are well studied, less is known about how beekeeping as a social practice adapts. This systematic review synthesizes 27 peer-reviewed studies across regions and disciplines, guided by social practice theory. We examine five interrelated dimensions—competences, materials, meanings, temporalities, and more-than-human relations—shaping beekeeping under climatic stress. Findings show adaptation is not limited to technical fixes. It includes changes in knowledge, hive design, timing, and shifts in cultural meanings and relations with bees and landscapes. Strategies range from hive modifications and transhumance to community learning and new framings of risk. However, adaptive capacity is uneven. Conceptualizing beekeeping as a climate-sensitive practice advances inter- and transdisciplinary dialogue and fosters more inclusive and effective adaptation strategies. This approach to reviewing practical climate micro-adaptation is replicable in other fields of practice.

## Introduction

Climate change is pervasively affecting how humans (and non-human species) carry out everyday practices, and in this review, we take stock of the growing scientific interest in climate impacts on bees and beekeeping (Decourtye et al. 2019; Neumann and Straub 2023; Zapata-Hernández et al. 2024). However, comparatively little attention has been paid to *beekeeping as a social practice*—one co-produced by skilled practitioners, bees, and their natural environment and carried out within communities of practice, and in constant attunement with more-than-human communities (Bremer et al. 2024; Remotti et al. 2024). Instead, existing studies mostly concentrate on the effects of climate change on bees as insects, on the plants they pollinate, or on plant–pollinator interactions and in particular focus on material (e.g. innovations in hive construction) and more-than-human dimensions (e.g. the selection of plants for regionally adapted flowering bridges, and the breeding of more varroa-resistant bees). Taken together, this constitutes a significant gap. Accordingly, our review focuses on how climate change is affecting beekeeping as a social practice and how these practices cope, adapt, and transform. In this context, social practice theory offers a promising interdisciplinary framework for understanding climate adaptation as a practice.

Assembling studies from all over the world enables a nuanced analysis of the adaptation strategies that are relatively common to beekeepers everywhere, e.g. securing hives against extreme weather—and those unique repertoires of action that beekeepers tailor to particular environments, e.g. through transhumance cycles that follow agricultural irrigation cycles. Our paper thus responds to calls in the literature for more social science research on the actual practices of beekeeping to address environmental challenges (cf. e.g. Patel et al. 2020; Fedoriak et al. 2021; Shanahan 2022), and climate change in particular (cf. Neumann and Straub 2023).

We use *social practice theory* (Reckwitz 2002; Shove et al. 2012) to construct the analytical categories of our systematic review paper (Hulme 2018). Inspired by Shove et al. (2012, p. 21), we understand beekeeping as a social practice composed of elements (competences, meanings, and materialities) that come together when the practice is carried out. Such a practice emerges, persists, or fades as the links between its defining elements are (re)formed or disrupted by climatic change. Departing from Shove et al. (2012), we add more-than-human agency and temporality as additional elements of the social practice of beekeeping, since this practice can only be accomplished in coordination with bees and other species, requiring that beekeepers synchronise practices to patterns of environmental rhythms and processes. Such a review of the bee(keeping) literature has not been done before. Furthermore, we assert that employing a social practice-inspired analytical framework could prove fruitful as a way of reviewing practical micro-adaptation to climate change in other fields of human activity than beekeeping.

Drawing on a broad interdisciplinary spectrum of research spanning rural and environmental studies, conservation research, apicultural and insect studies, as well as climate research, we identified peer-reviewed papers that examine the impacts of climate change on beekeeping practices and the ways in which beekeepers adapt. Section "Materials and methods" outlines the methodology used to select the text corpus for this systematic literature review. Section "The Text Corpus" then maps this corpus and examines how the reviewed studies conceptualise climate change in relation to beekeeping, as well as the methods employed to study beekeeping practices. Section "Social Practice Theory" introduces social practice theory, which informed the selection of the analytical categories used in this paper. Building on this framework, Section "Analysis" analyses the text corpus along five conceptual dimensions: competences, materials, meanings, temporalities, and more-than-human perspectives. Section "Discussion" examines the implications of this systematic literature review for beekeeping in agricultural and socio-ecological contexts and assesses the usefulness of social practice theory for analysing micro-level adaptation processes to climate change. The concluding section synthesises the main insights of the review and outlines broader implications for future research at the interface of beekeeping, climate change, and social practice approaches.

## Materials and methods

**Fig. 1:**
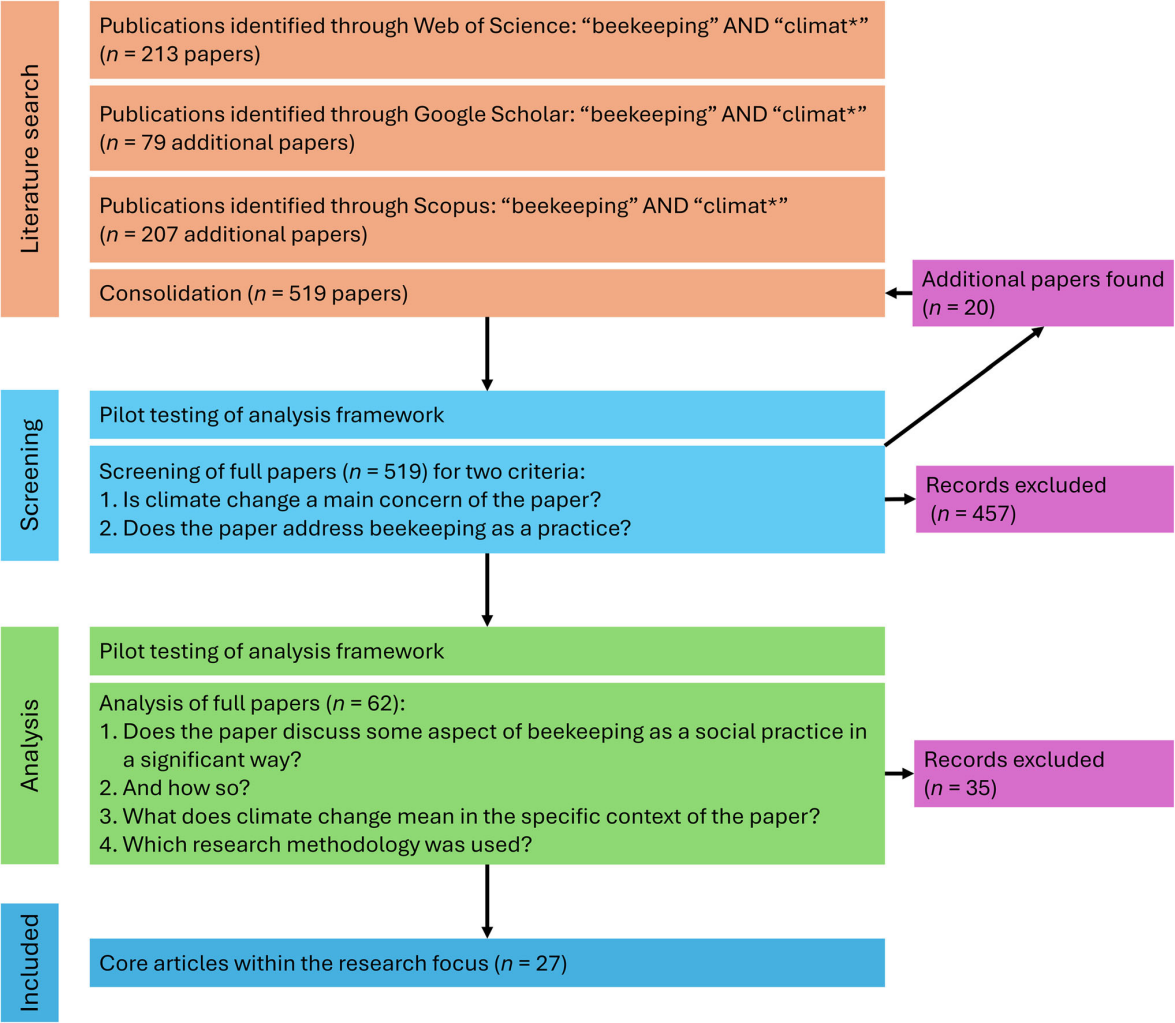
Flow chart of the systematic literature review process

In this systematic literature review (Feak and Swales 2011), we examine how the scientific literature addresses the ways in which beekeeping—as a social practice—is affected by climate change and how it is adapting to or coping with its impacts. Our analysis is guided by social practice theory, from which we derived the analytical categories used in the review (for an overview of the systematic review process, see Fig. 1).

We employed the broad search terms “beekeeping” AND “climat*” to conduct a scan of titles, abstracts, and keywords across three major databases: Web of Science, Google Scholar, and Scopus. This search yielded 213 English-language publications in Web of Science, a further 79 in Google Scholar, and an additional 207 in Scopus. Screening reference lists during the review process, we identified a further twenty potentially pertinent papers. At later stages of analysis, no additional publications (prior to May 2024) were retrieved. Thus, our initial, unrefined corpus comprised a total of 519 English-language articles.

The papers were reviewed in two rounds. An analysis template was developed and tested for each round. The authors responsible for the review (SM, SB) initially analysed the same papers to synchronise their analysis. After this test run, the corpus was divided equally. In the first round, two criteria were considered: firstly, whether climate change was a main concern in the paper (and not just mentioned as one of many other environmental issues) and in what way; and secondly, whether the paper addressed beekeeping as a practice.

Consequently, papers on beekeeping practices such as honey hunting in certain regions of Africa and Southeast Asia were also included in the text corpus (Chantawannakul 2018; Paraïso et al. 2012), as they met the inclusion criteria—a primary focus on climate change and the discussion of a form of beekeeping as a social practice. However, we are aware that these practices possess a significantly different character. Therefore, in our analysis, these papers were considered cautiously. Nevertheless, their inclusion proved to be productive, as they offered valuable insights into the social practice of beekeeping in times of climate change.

In turn, papers that discussed the effects of climate change on phenology (cf. e.g. Schweitzer et al. 2013; Stemkovski et al. 2020; Prado et al. 2021), plant-pollinator interactions (cf. e.g. Petanidou 2003; Klein et al. 2007; Hegland et al. 2009; Reddy et al. 2012; Rafferty 2017; Knight et al. 2018), or bee wellbeing (cf. e.g. Le Conte and Navajas 2008; Brown and Paxton 2009; Menzel and Feldmeyer 2021) were excluded if they did not elaborate on how these affected the social practice of beekeeping. Similarly, papers that discussed beekeeping as a social practice but did not clearly link this to climate change (adaptation) were excluded (cf. e.g. Phillips 2014, 2020; Maderson and Wynne-Jones 2016; Adams 2018; Cilia 2019; DiDonato and Gareau 2022; Mouillard-Lample et al. 2023). Finally, papers that advocated beekeeping as a strategy to increase the climate resilience of smallholder farmers or sustainable development in general were also removed if these did not demonstrate a link to the practice of beekeeping (cf. e.g. Unks et al. 2019; Mngumi 2021; Mpondo et al. 2021; Patel et al. 2021; Pocol et al. 2021; Schouten 2021; Lazos-Chavero et al. 2022; Shadrack and Mwalilino 2022).

After these exclusions, the final corpus consisted of 62 papers. In the second round, the research methodology and analytical approach of the papers were reviewed—is it research with, of or relevant to beekeepers?—and, as selection criteria, whether they addressed the aspects of beekeeping as a social practice that we had distilled from the theoretical literature (Reckwitz 2002; Shove et al. 2012). After the second round, the final text corpus consisted of 27 papers.

The inclusion of less than ten per cent of the initial text corpus warrants some clarification. This outcome is partly attributable to our intentionally broad selection of search terms, which yielded not only publications explicitly addressing climate change, but also those concerned with climatic zones, microclimates within beehives, and the social climate of beekeeping—topics that fall outside the scope of our review. Our rationale for employing such inclusive terms was to minimise the risk of overlooking potentially relevant literature. At the same time, this outcome underscores the relative scarcity of research on climate change adaptation in beekeeping as a co-produced social practice.

## The text corpus

### Background of the text corpus

**Fig. 2:**
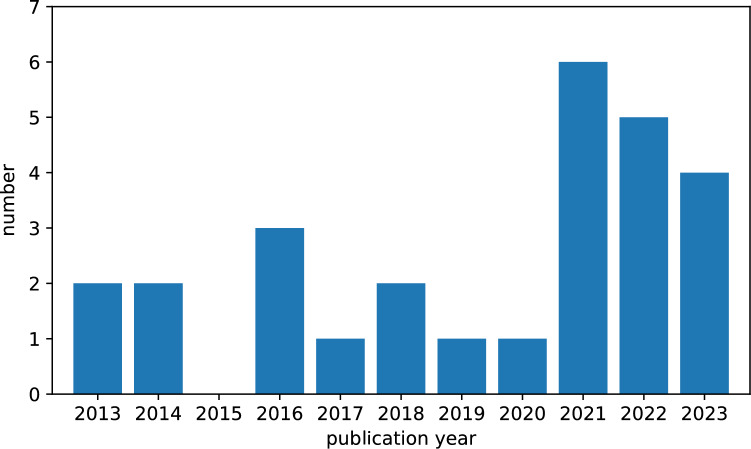
Publication dates of papers in the text corpus

Our review reveals an increase in research on the impacts of climate change on beekeeping, particularly from 2021 onwards, as shown in Fig. 2. This surge highlights the escalating concern within the scientific community as environmental challenges intensify. It may also reflect a growing interest in practices and human-nature relations in the environmental social sciences and humanities.

With regard to geographical distribution, most case studies focus on Africa (n = 10) and Europe (n = 9), whereas North America (n = 4) and South America, Australia, and Asia (each n = 1) are less frequently represented, suggesting regional differences in research priorities and beekeeping practices.

**Fig. 3:**
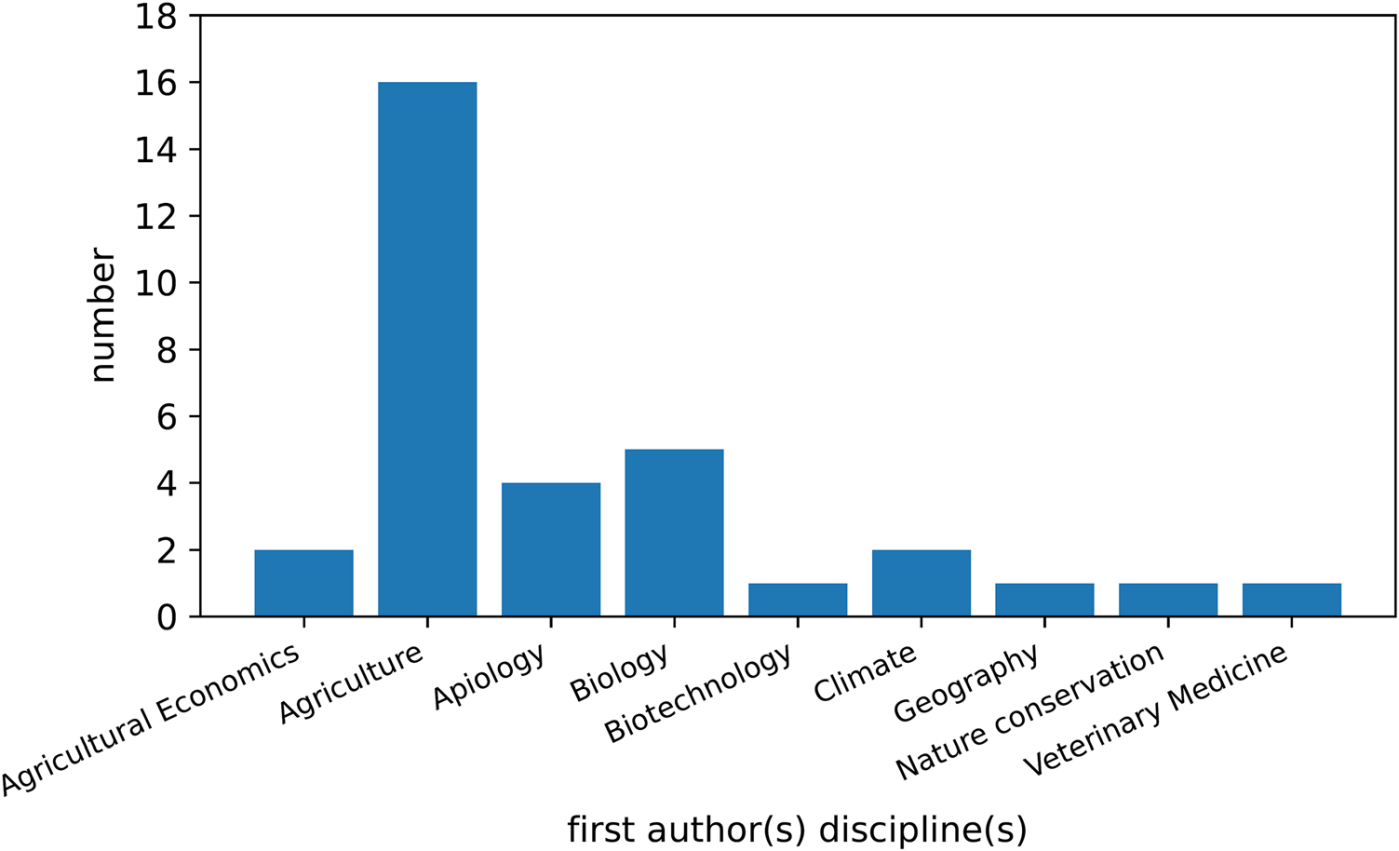
Disciplinary background of main authors

As shown in Fig. 3, most of this research is authored by experts in agriculture, with additional contributions from fields like apiculture science and environmental science. This suggests that the research is largely viewed through an agricultural lens, emphasizing practical impacts on beekeeping. To categorize authors by scientific discipline, we used the first author(s) affiliations provided in the papers.

### Methods used in the text corpus

In the text corpus, different methodological approaches were used to capture beekeepers’ experiences and knowledge. There were four desktop studies (Hoover and Hoover 2014; Abou-Shaara 2016; Chantawannakul 2018; Decourtye et al. 2019) and two experimental designs (Calovi et al. 2021; Cebotari and Buzu 2022). In addition, there were two studies based on interviews (Lehébel-Péron et al. 2016; Landaverde et al. 2023), four based on questionnaires or surveys (Kumsa 2014; Gallardo-López et al. 2021; El Agrebi et al. 2022; Kuliçi et al. 2023) and three using a combination of the two (Aynalem 2017; Gajardo-Rojas et al. 2022; Van Espen et al. 2023). Twelve papers used focus groups, either as the only method (Fedoriak et al. 2021; Novelli et al. 2021; Vercelli et al. 2021), in combination with interviews (Berhe, et al. 2013; Degu 2022; Degu et al. 2022; López-i-Gelats et al. 2022), or with interviews and observation methods (Paraïso et al. 2012; Mtengeti et al. 2013; Malisa and Yanda 2016; Dimelu and Eberechukwu 2018; Patel et al. 2020).

### Representations of climate change

How is climate change represented by authors in the corpus? Given their focus, papers framed climate change by its impacts on bees and beekeeping, both direct and indirect. Direct impacts include rising temperatures, which impact bee health and productivity. More frequent extreme periods with heat stress increase colony mortality, reduce foraging activity and honey production. Other extreme weather events such as storms and floods can damage apiaries, stress the colonies, and negatively affect their survival rates (Paraïso et al. 2012; Dimelu and Eberechukwu 2018; Vercelli et al. 2021; Degu 2022; Gajardo-Rojas et al. 2022; Van Espen et al. 2023).

Indirect impacts were described in terms of changes in vegetation patterns resulting from climate change, which alters the availability and diversity of forage plants, reducing the nutritional quality and quantity of bee food sources. More variable rainfall patterns disrupt flowering times, potentially leading to mismatches between bee activity and floral resource availability (Paraïso et al. 2012; Malisa and Yanda 2016; Dimelu and Eberechukwu 2018; Vercelli et al. 2021; Degu 2022; Gajardo-Rojas et al. 2022; Van Espen et al. 2023). Droughts dramatically affect the vegetation and limit nectar and pollen supply, decreasing honey production and increasing the risk of colonies starving (Aynalem 2017; Degu 2022; Gajardo-Rojas et al. 2022). The spread of pests and diseases (such as such as Varroa destructor, Nosema ceranae, the Asian hornet, greater wax moth, small and large hive beetle) are also exacerbated by warmer winters and altered environmental conditions, increasing colony losses, and necessitating frequent and costly treatments (Calovi et al. 2021; Degu 2022; El Agrebi et al. 2022; Kuliçi et al. 2023; Van Espen et al. 2023; Vercelli et al. 2021).

Related to indirect impacts, papers highlighted some factors that contribute to, or compound, impacts. Pesticide use, often intensified by climate change-driven pest pressure, directly harms bee populations through exposure and habitat degradation (Paraïso et al. 2012; Aynalem 2017; Dimelu and Eberechukwu 2018; Novelli et al. 2021). Land-use change, including deforestation and habitat loss from monoculture farming spurred as a climate risk response, further reduces the availability and diversity of forage plants, exacerbating the impacts described above (Chantawannakul 2018).

## Social practice theory

This study distinguishes itself from other ‘bees and climate’ reviews (Decourtye et al. 2019; Neumann and Straub 2023; Zapata‐Hernández et al., 2024) with its focus on how climate change is impacting on *beekeeping as a social practice*, and what beekeepers as a group are doing differently to adapt to these impacts. It enables a comparison of the *doing of* beekeeping in different environments worldwide, helping to draw lessons and highlight gaps in adaptation research and practice. To this end, the review focuses on the culturally routinised behaviour of beekeepers—their patterns of thinking, doing, and feeling with material things—rather than on the agency of individual beekeepers or the social institutions they work within (though agency and institutions do influence practice—see Bremer et al. 2024; Phillips 2020). This focus is also a function of the data available in scientific papers, which by convention tend to overlook individuals’ micro-scale decisions, or wider social contexts. To structure this focus, and systematically analyse elements of practice, we designed the review within the framework set by social practice theory (Reckwitz 2002; Shove et al. 2012).

**Fig. 4:**
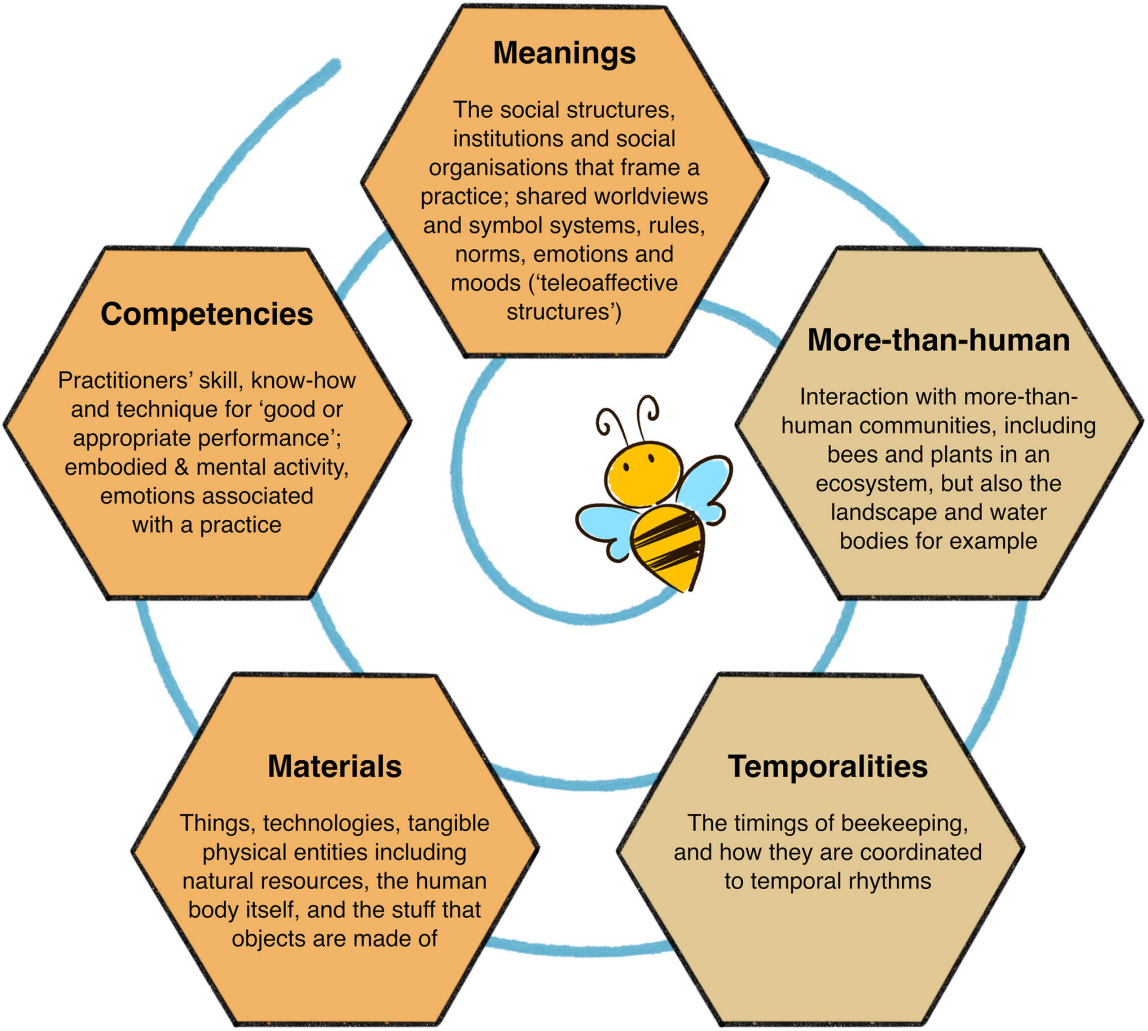
Social practice theory—elements of practice and their temporal and interspecies relations (based on Reckwitz 2002; Shove et al. 2012; credit: Anna Voigtländer)

Social practice theory comes from a family of approaches for studying culture in the everyday lived activities and interactions of social groups. It focuses on practices themselves as the unit of analysis, with Reckwitz (2002, p. 250) defining practices as, ‘a routinised way in which [human] bodies are moved, objects are handled, subjects are treated, things are described, and the world is understood’. Far from random actions, practices are ‘enduring entities reproduced through recurrent performance’ by people as ‘carriers’ (see practitioners) who carry out a routinised pattern of action (Shove et al. 2012, p. 8). Beekeeping, for instance, has ancient roots, and beekeepers today adhere to several standardised approaches that are picked up by groups worldwide. Yet, while enduring, practices are also in constant evolution through a dynamic interaction between elements; with each ‘performance’ a practitioner adjusts how they carry out an activity, ‘linking new and existing elements’ (Shove et al. 2012, p. 15). Consider how a beekeeper may try out a new technique for housing the bees or install a new technology like a sensor, or how a group may decide to move their hives and so initiate a pattern of transhumance. This makes the theory well suited to analysing how beekeeping practices adapt—cope and transform—as conditions change with climate change. Scholars variously break down the elements of practice, but three elements feature prominently in conceptualisations (Reckwitz 2002; Shove et al. 2012)—(i) meaning; (ii) competences; and (iii) materials—which we drew on to analyse beekeeping (see Fig. 4). Inspired by them, we see beekeeping as a social practice made up of these three elements that are integrated when a practice is enacted. Beekeeping as a social practice emerges, persists, and disappears as links between its defining elements are (re)made or broken in the face of climatic change.

Our review also wanted to highlight two relational characteristics of (beekeeping) practices that are vital for understanding climate impacts. First, the relation of practices to time, beyond considerations of how practices persist over time (Shove et al. 2012). Practices take time, and we can see time as a limited resource in a day (or week, or season) when people are active. Concurrently, practices create a sense of time, insofar as they set a tempo to social life, and make visible certain social and environmental temporalities important for a practice. And practices are timed, meaning that there are opportune moments for carrying out a practice—e.g. moving beehives to an orchard for pollination—and appropriate ways for sequencing and ordering practices. We can see practices as coordinated to patterns of rhythms—e.g. rhythms of the bee colony, flowering, weather, or agricultural pesticide use and honey market—and that these rhythmic patterns are being disrupted by climate change. Second, and related, beekeeping practices are accomplished at the intersection of the ‘activities and processes’ of multiple more-than-human communities. Most directly, it demands a form of partnership between human beekeepers and bees, with both species aligning to each other’s activities and timings. But the practice also implicates other living species, of flowers for example, and can even be extended to the dynamic processes of more-than-human communities within a whole landscape (consider processes of erosion, or water bodies for instance).

## Analysis

This chapter examines how the literature in our text corpus addresses the ways in which climate change challenges and reconfigures the elements of beekeeping as a social practice—meaning, competence, materials, more-than-human agency, and temporality—as well as the links between them. While we recognise the global diversity of beekeeping practices and do not aim to synthesise or homogenise them, our analysis is based on the premise that they reflect different configurations of these shared elements. We are particularly interested in how the academic literature portrays the effects of climate change on each of these elements and their links.

### Meaning

Globally, bees and honey are culturally embedded and thus co-determine the practice of beekeeping and the social role of beekeepers. Looking at Southeast Asia, Chantawannakul (2018, p. 169) explains: ‘Honey bees are social insects that not only produce food but also play an important part in religious beliefs and cultures’. Honey is not only a food (e.g. sweetener), but also used for medical purposes (human and veterinary) or as a commodity in barter trade (e.g. Mtengeti et al. 2013; Chantawannakul 2018). Religious beliefs play a role when it comes to climate change adaptation as Paraïso et al. (2012, p. 529) found out for beekeepers in Benin, for whom prayer practices are the second common adaptive strategy. Meanwhile, beekeeping offers many people in the Global North a way to understand their socio-natural environments, e.g. by reading changes in flowering phenology and honey yields as signals of shifting climate and land-use conditions, as shown by Italian beekeepers or heather honey beekeepers in southern France (e.g. Lehébel-Péron et al. 2016; Malisa and Yanda 2016; Decourtye et al. 2019; Novelli et al. 2021).

As a social practice, beekeeping comes with different ends and aspirations. For many beekeepers, it is the main or an important additional source of household income (Paraïso et al. 2012; Fedoriak et al. 2021; Degu 2022; Degu et al. 2022; Van Espen et al. 2023). For many, it takes more the role of a hobby, rest, art, or a tool for self-realization (Fedoriak et al. 2021, p. 770). Others relate it to some environmental mission such as ‘trying to save their bees from the detrimental effects of pesticides by transporting their apiaries to cities’ (Fedoriak et al. 2021, p. 770) or contributing to nature conservation efforts (Novelli et al. 2021; Vercelli et al. 2021). Honey sorts—e.g. heather honey in southern France (Lehébel-Péron et al. 2016)—can create a kind of regional identity that beekeepers want to uphold in a changing climatic and market situation.

Beekeepers’ approach to climate change is strongly influenced by their motivation to engage in beekeeping in the first place. It seems that the alertness to climate change, the perception of risk, the ability to adapt, and the choice of adaptive strategy differ among professional and hobby beekeepers as ‘professional beekeepers may regard that there are fewer possibilities for adaptation, since they are already operating at a maximum capacity, at a certain optimum or limit, or with a minimal margin. […]. By contrast, hobbyists—who are not dependent on beekeeping as their main or sole source of income—can more easily scale up or down, or even drastically change their beekeeping management practices, even though this might go hand in hand with an extended period of lower (economic) performance or honey yields’ (Van Espen et al. 2023, p. 9, cf. also El Agrebi et al., 2021; Gallardo-López et al. 2021; López-i-Gelats et al. 2022).

In many regions worldwide, climate change is considerably adding to the stress beekeepers are already witnessing. However, also the degree of stress and coping strategies, e.g. which materials to use, which actions to perform and when, or which knowledge and competence to acquire varies globally and regionally within areas. Generally, beekeepers in parts of Africa, Latin America and Southern Europe already feel much more negatively affected by climate change than those in Northern Europe (cf. e.g. Paraïso et al. 2012; Abou-Shaara 2016; Gajardo-Rojas et al. 2022; Van Espen et al. 2023). In the face of climate change, many feel helpless and economically unable to adapt (cf. e.g. Paraïso et al. 2012; Gallardo-López et al. 2021; Van Espen et al. 2023). Fear of poverty and the wrong adaptive choices cause them to abandon their business or overuse the bee colonies (Paraïso et al. 2012; Gallardo-López et al. 2021).

The text corpus identifies two strategies to help beekeepers adapt better to climate change and to give new meaning to the different elements of beekeeping practices. Firstly, beekeepers’ spirit and strong motivation as well as their sense of (intergenerational) community is seen as a great strength of the sector (Patel et al. 2020; Vercelli et al. 2021; cf. also Novelli et al. 2021). Especially where beekeepers are not yet organised and do not discuss adaptation strategies, such unions are seen as a great opportunity (cf. e.g. Dimelu and Eberechukwu 2018; Kuliçi et al. 2023; Mtengeti et al. 2013). Secondly, and relatedly, beekeepers can learn how to deal with the challenges of climate change. This can be achieved informally by peer-to-peer learning or through beekeeping associations, but also through other educational contexts. Both strategies are considered to have the potential to influence teleaffective structures so that beekeeping practices can be meaningfully maintained in times of climate change.

### Competence

Beekeepers around the world are experiencing how climate change affects their competence as practitioners. As a result, they are changing what they do and when they do it (e.g. Abou-Shaara 2016; Van Espen et al. 2023), acquiring new knowledge and adopting new skills and techniques (Decourtye et al. 2019). This applies to virtually all aspects of beekeeping, such as pest control (Hoover and Hoover 2014; Kumsa 2014; Abou-Shaara 2016; Decourtye et al. 2019; Novelli et al. 2021; Calovi et al. 2021; El Agrebi et al. 2022), feeding (Kumsa 2014; Decourtye et al. 2019; Degu et al. 2022), transhumance (Paraïso et al. 2012; Dimelu and Eberechukwu 2018; Decourtye et al. 2019; Novelli et al. 2021), harvesting (Kumsa 2014; López-i-Gelats et al. 2022) or preparing bees for the winter (El Agrebi et al. 2022; Novelli et al. 2021). Hive management in general is changing (Kumsa 2014; Abou-Shaara 2016; El Agrebi et al. 2022; López-i-Gelats et al. 2022) as ‘beekeeping may become more challenging, more time-consuming, and will require additional skills and efforts from beekeepers. For example, […] the likelihood that honey bee colonies will need to be monitored all year round and checked more frequently, particularly with regard to food stocks, newly emerging pests and disease infestation’ (Van Espen et al. 2023, p. 5). New skills include the ability to cultivate climate-adapted bee pastures whose plants will bridge periods of low pollen and nectar flow (Section “Interaction with the more-than-human”) (Abou-Shaara 2016; Dimelu and Eberechukwu 2018; Decourtye et al. 2019; Degu et al. 2022).

In this changing environment, learning plays a crucial role. Through their hive records, beekeepers have a vast source of local ecological knowledge of local climate variability and change to share (Lehébel-Péron et al. 2016, p. 133). Beekeeping appears to be a practice that is passed on within families. A study on Australian beekeeping elaborates that ‘[i]ntergenerational beekeepers followed the knowledge of their parents and grandparents regarding the rich spatial–temporal history of resources, production, weather, and issues at their regular forage sites, and were also involved in sharing beekeeping knowledge by training new beekeepers’ (Patel et al. 2020, p. 23; cf. also Degu et al. 2022). Learning also takes place through ‘mutual collaboration among beekeepers’ (López-i-Gelats et al. 2022). So, while beekeeping seems to be a practice that is successfully learned within the community of practice, some studies note that the lack of more formalised ways of acquiring competence is seen as a barrier to better adaptation to climate change (Mtengeti et al. 2013; Dimelu and Eberechukwu 2018; Novelli et al. 2021; Degu et al. 2022; Kuliçi et al. 2023). For instance, an interviewee in a European project believes that ‘climate change will be a selection criterion for beekeepers [rather than the bees]: those who do not have enough knowledge will run out of bees and give up’ (Van Espen et al. 2023, p. 5). This includes, also for established beekeepers, the ability to integrate into their practices available information technology, from online weather data to sensor-based hive management techniques (Novelli et al. 2021).

### Materials

Materials play a crucial role in beekeeping. The basic idea of this social practice is the same all over the world: Beekeepers need some kind of container to house the bees, and at some point, they will control pests, harvest the honey, and prepare the bees for the flower-scarce period, typically winter, when nectar and pollen availability is low. However, depending on the materials used, the practice of beekeeping varies considerably. For example, there are different sizes and shapes of hives, which require specific frames (or no frames at all) and specific tools. Or, if beekeeping is for honey production, pollination services or both, more or different materials are needed.

Climate change leads to a reconfiguration of the material elements of beekeeping. For example, beekeepers modify their hives to cope better with extreme weather conditions (more heat, strong winds and rain) (Paraïso et al. 2012; Berhe, et al. 2013; Degu et al. 2022; Landaverde et al. 2023). Climate change brings higher temperatures and changes in vegetation to which beekeepers must adapt. As a result, new materials come into play or existing ones become more relevant. Beekeepers create objects for shading and cooling, such as stone bunds (Aynalem 2017; Degu et al. 2022) or by planting shade trees (Dimelu and Eberechukwu 2018). Changes in vegetation affect nectar and pollen sources for bees, so creating bee pastures by introducing new and more climate-resistant plants is seen as a solution (Aynalem 2017; Chantawannakul 2018; Decourtye et al. 2019; Degu et al. 2022), though this requires beekeepers to have access to significant tracts of land (Degu et al. 2022). Feeding, e.g. with sugar pads, becomes more important than before, as food sources become scare or unreliable (e.g. Chantawannakul 2018; Decourtye et al. 2019; Degu et al. 2022). To find cooler and/or more nectar-rich areas, many beekeepers practice transhumance (e.g.Gallardo-López et al. 2021; Gajardo-Rojas et al. 2022), for which they need the means of transport, but also the financial resources to cover the higher costs, especially for fuel (Patel et al. 2020; Gallardo-López et al. 2021; López-i-Gelats et al. 2022). Beekeepers also use new information technologies to track the weather (Patel et al. 2020).

Honey is a key material element of beekeeping. Many beekeepers worldwide are experiencing declining honey yields (Kumsa 2014; Lehébel-Péron et al. 2016; Decourtye et al. 2019; Degu 2022; Degu et al. 2022; Gajardo-Rojas et al. 2022). In addition, others ‘jump-over harvesting (indirectly feeding the bee colonies with the existing hive resources of honey and pollen) rather than buying supplementary feed (such as sugar and bean flour)’ (Degu et al. 2022, p. 1038). To sell their honey better, beekeepers need economic tools such as a credit system (Dimelu and Eberechukwu 2018) or other marketing tools (Lehébel-Péron et al. 2016). Meanwhile, to compensate for lower honey production, beekeepers run the risk of putting too much pressure on colonies, thus hindering their regeneration (Paraïso et al. 2012).

Research in the corpus also treats bees as a material element of beekeeping. Like other materials, they can be modified, for example through breeding techniques that promise to produce more climate-adapted bees (Hoover and Hoover 2014; Abou-Shaara 2016; Gallardo-López et al. 2021). Others suggest using a greater diversity of bees for pollination or even robotic bees (Hoover and Hoover 2014; Decourtye et al. 2019). Climate change affects bee health through heat stress or extreme weather conditions, but also by creating conditions favourable to the spread of pests such as the Varroa mite and invasive species such as the Asian hornet. Beekeepers therefore use new or more materials (such as chemicals or hive modifications) to treat and protect bees from these challenges (Decourtye et al. 2019; El Agrebi et al. 2022). In this review, we added the more-than-human element to capture the agency of bees as ‘collaborators’ in beekeeping, and not simply as materials, like wooden hives or sugar (Section “Interaction with the more-than-human”).

### Interaction with the more-than-human

Key to beekeeping is how it is accomplished in conjunction or attunement with the behaviours and rhythms of multiple more-than-human communities. Bees, humans, and the wider ecosystems they dwell within have co-evolved relative to each other over millennia. The reciprocal relation between Apis mellifera (the European honeybee) and Homo sapiens is central here, having co-adapted over 15.000 years (Lehébel-Péron et al. 2016). But bees and beekeepers also interact with other species in an environment (Matias et al. 2017); from the plants that bees forage, to the animals and insects that predate on bees. And as environments change, these more-than-human communities adapt—their distribution, behaviours, and timings—demanding that beekeepers too adapt to keep in step.

Papers in the corpus discussed shifts in bees’ behaviours relative to environmental change. Bees are coping with extreme heat by shortening forage flights and altering routines for reducing hive temperatures, through ‘fanning’ behaviours or where they build their wild-hives for example (Abou-Shaara 2016; Chantawannakul 2018; Landaverde et al. 2023). This sees shifts in the seasonality of the hive, with changes in when colonies take their cleaning flight, or swarm for instance (Vercelli et al. 2021; Cebotari and Buzu 2022). With increased heat, hives need more water and food, but several scholars saw these resources becoming scarcer, demanding that bees spend more time and energy looking for them (Vercelli et al. 2021; Landaverde et al. 2023). Taken together, beekeepers note that hives are coming to produce less honey, have a lower density of bees, have less fertile queens, and ultimately a higher chance of collapse (Calovi et al. 2021; Vercelli et al. 2021; Landaverde et al. 2023). In the long term, authors discuss how bees will evolve (Abou-Shaara 2016), and debate which species will prove more resilient, native bees or honeybees (Hoover and Hoover 2014).

Climate change is affecting flowering plants’ behaviour, with implications for bees’ forage. The temporal distribution of flowerings over the year is reportedly changing, variously discussed as early or late flowerings, and flowering periods that are compressed or stretched in ways that make flowering ‘uneven’ or even random (Abou-Shaara 2016; Patel et al. 2020; Cebotari and Buzu 2022). Van Espen et al. (2023, p. 9) discuss this as the ‘flowering arch’ that bridges the season; ‘diverse plants and trees with different flowering periods from early on till late in the bee season [which is] becoming stretched, less predictable and/or less well synchronised with developments and foraging activities of honey bee colonies’. Environmental change also sees an altered distribution of flowering plants, and the spread of invasive plants with lower levels of pollen (Chantawannakul 2018; López-i-Gelats et al. 2022). Indeed, some authors report reduced nectar in all local wildflowers (Landaverde et al. 2023).

Aynalem (2017, n. p.), in turn, stress that ‘[h]oneybees need to adapt to the entire array of pests, predators, and parasites and pathogens and chemicals surrounding them because of climate change’. Warmer temperatures and high humidity affect the behaviours of pests that affect the hive, introducing new pests (e.g. small hive beetles) or new preditors (e.g. bee-eater birds) (Aynalem 2017, n. p.), or rendering the bees more vulnerable to attack. Landaverde et al. (2023, p. 6) noted that, ‘[Varroa] affects honey bees more in scorching seasons, perhaps because they are even more stressed and have higher work demands’.

A relational regard for the more-than-human also sees practices in the larger context of a landscape, and how landscapes are altered through land use change and agrochemicals. Authors noted how expanded agricultural land, often in monocropping, and urbanization has reduced forest cover and biodiversity, affecting bees’ forage (Chantawannakul 2018; Decourtye et al. 2019). Climate change is affecting which crops agriculturalists choose to grow, with implications for the food available to bees (Aynalem 2017; Degu 2022). A changing climate is also increasing pest numbers in some locations, leading agriculturalists to increase their use of pesticides and broad-spectrum herbicides, which have proven deadly to bee colonies (Aynalem 2017; Decourtye et al. 2019).

### Temporalities and timings

Understanding the evolution and adaptation of beekeeping practices means understanding the dynamic relations between beekeeping materials, meanings, competences, and more-than-communities, in and over time. Indeed, temporal themes appeared in almost all papers. Insofar as time represents a limited resource for carrying out an activity—beekeeping takes time—several authors described climate change as altering the time available; ‘the main impacts […] will be in relation to food resource availability, changing local weather conditions, the length of the bee season” (Van Espen et al. 2023, p. 4). While some saw the season lengthening with shorter periods of cold weather, others saw how erratic patterns of extreme weather or varroa infestation, can interrupt the season.

Beekeeping also creates a sense of time; it makes visible the temporal rhythms that beekeeping is entwined with, which sets a tempo and order to the practice. Papers discussed how climatic change is affecting cycles in the environment, especially to weather and hydrological patterns (Abou-Shaara 2016; Degu 2022; Gallardo-López et al. 2021). This was discussed as shifts in precipitation (the amount, timing, and reliability), increasing temperatures (summer stress on hives, unstable winters), and an increase in extremes (droughts, cyclones, rainstorms) (Lehébel-Péron et al. 2016; Novelli et al. 2021; Gallardo-López et al. 2021; Landaverde et al. 2023). By extension, authors saw disruption to river flow and flooding, the water resources available to bees, and soil moisture (Lehébel-Péron et al. 2016; Gajardo-Rojas et al. 2022; Landaverde et al. 2023). Altered climatic rhythms are reflected in altered phenological cycles, with plants flowering earlier, more erratically or for shorter periods (Decourtye et al. 2019; Cebotari and Buzu 2022; Gajardo-Rojas et al. 2022), affecting the ‘arch’ of flowering plants that bridge the season (Van Espen et al. 2023). Extreme events also affect when bees access food resources, with storms and extreme rain damaging flowers, or droughts withering plants (Degu 2022). Climatic change is also affecting the distribution of plant species and habitat, with implications for the ‘timescape’ (Adam 2018) that beekeepers navigate by (López-i-Gelats et al. 2022). When patterns of environmental rhythms are disrupted, authors note the trophic mismatches that result (Aynalem 2017; Calovi et al. 2021). As an interviewee reported in Van Espen et al. (2023, p. 5) observes; ‘Bees are regulated by temperature, but this year we had bees flying in January. [But] bee activity and plant flowering should be correlated. What we see now is that biological calendars are no longer synchronised’.

Authors wrote about how colonies are adjusting the timings of the hive to attune to altered environmental rhythms (Abou-Shaara 2016; Degu 2022; Landaverde et al. 2023). In the everyday, this affects when and how long bees can forage (e.g. under extreme heat), and their physiological need for water. Seasonally, this relates to how the hive times activities over the year, with Cebotari and Buzu (2022) noting for example how bees in Poland are performing their ‘cleansing flight’ (following hibernation) a full month earlier than the average over the past 25 years. Warmer climates and altered colony behaviour can also result in increased disease levels, with longer periods of brood rearing leading to higher levels of varroa for instance (Calovi et al. 2021). As an interviewee reported in Van Espen et al. (2023, p. 5) notes, ‘Pathogens and parasites are not freezing to death anymore because of the lamentable winters we are having’.

Finally, beekeeping practices are ‘timed’, meaning actions are adapted to synchronise to opportune moments in the temporal patterns beekeepers (and bees) track. For beekeepers, this demanded that they change their timings of inspecting and preparing their hives for the bee season (Kumsa 2014; Abou-Shaara 2016), increased varroa mite control treatments including practicing winter-season ‘biotechniques’ (Novelli et al. 2021), and feeding colonies over periods when flowering fails, or alternatively skipping harvesting of honey to let the colony consume it (Kumsa 2014; Degu et al. 2022; Van Espen et al. 2023). ‘Beekeepers agreed that the most important action related to the lack of nectar is supplying the colonies with supplemental feeding […] in periods with no flowering and low temperatures, such as in late autumn and winter’ (Vercelli et al. 2021, p. 5). Other adaptation techniques involve manipulating the temporalities of the landscape. This could be through planting species of flowers with variable phenology to rebuild the ‘flower arch’ (Decourtye et al. 2019). Or through initiating patterns of transhumance; moving hives around the landscape to chase ephemeral water and food resources (Novelli et al. 2021; Gajardo-Rojas et al. 2022). This includes synching with other activities in the landscape, for example by moving hives close to irrigated areas during dry spells (Vercelli et al. 2021).

## Discussion

### Beekeeping as a climate-sensitive social practice

In this review, we used social practice theory as a framework for making visible how beekeepers are adapting their practices. We find that climate change affects the configurations through which meanings, competences, materials, temporalities, and more-than-human relations are integrated in practice. This sees beekeeper communities working—individually and collectively—to reconfigure practice elements. For example, engaging in transhumance to access distributed pollen and nectar sources requires new skills, materials, and timings. The success of such adaptations depends on whether these elements can be effectively realigned.

At the same time, our review highlights limits to adaptation. Some risks threaten to disrupt entire practices, not least extreme heat stress on hives or declining social and financial capital among beekeepers. Honey hunting provides an illustrative example. Here, the combined effects of climate change on vegetation, together with changing patterns of agricultural land use and urbanisation, appear to be steadily shrinking the habitats available to wild bees, while continued extraction places further pressure on bee populations. In consequence, the links between the elements sustaining honey hunting as a social practice may be at risk of breaking down. This observation is based on only two studies (Paraïso et al. 2012; Chantawannakul 2018) from different world regions (Southeast Asia and West Africa), indicating the need for further research. These limits to adaptation also point to pronounced differences in who can adapt, and under which conditions.

### Uneven adaptive capacity and differentiated meanings

A central finding of this review is that adaptive capacity is unevenly distributed. While beekeeping globally shares core elements, their configuration varies strongly across socio-ecological contexts. Our aim was therefore not to compare disparate forms of beekeeping directly, but to examine how climate change affects the elements of beekeeping as a social practice and how these elements are reconfigured—or, in some cases, disrupted.

Professional and hobby beekeepers, for instance, experience climate risks differently. Professional beekeepers—often operating close to economic and logistical limits—frequently perceive fewer viable adaptation options, whereas hobbyists may experiment more readily with altered management practices, even at the cost of lower yields. Similarly, beekeepers in parts of Africa, Latin America, and Southern Europe report far more acute impacts than those in Northern Europe, reflecting differences in climatic stress, access to resources, and institutional support.

Meanings attached to beekeeping play a crucial mediating role here. As shown in Section “Meaning”, beekeeping may be primarily a livelihood strategy, but also a cultural practice, hobby, or environmental commitment. These teleoaffective structures shape how climate risks are perceived, which adaptations are considered acceptable, and how much loss can be tolerated. Our synthesis suggests that adaptation strategies are more likely to persist when they resonate with beekeepers’ motivations and identities, rather than being framed solely as technical fixes.

Community cohesion and collective learning repeatedly emerge as enabling factors. Beekeeping associations, peer-to-peer learning, and intergenerational knowledge transmission help practitioners reinterpret climatic change, share experiential knowledge, and coordinate responses. Such social infrastructures are therefore integral components of adaptive capacity, even though they are rarely foregrounded in technically oriented research.

### Reconfiguring competences, materials, and timings beyond technical fixes

Climate change demands heightened temporal attunement from beekeepers, as altered flowering phenologies, extended or disrupted seasons, and increased climatic variability require more frequent monitoring, flexible scheduling, and revised sequences of action (Section “Temporalities and timings”). Beekeepers respond by changing inspection routines, adjusting harvest timings, skipping harvests altogether, or feeding colonies during unexpected forage gaps. Adaptation thus increasingly takes the form of temporal labour, as practitioners work to resynchronise their practices with shifting environmental rhythms (Bremer and Wardekker 2024).

At the same time, relations with more-than-human actors are being renegotiated (Section “Interaction with the more-than-human”). Bees respond to heat, drought, and pests by altering foraging behaviour, colony dynamics, and seasonal cycles. Beekeepers, in turn, must accommodate these changes, often with incomplete knowledge and heightened uncertainty.

### Methodological implications and social practice theory

These findings also raise methodological questions. Our review reveals a clear methodological pattern in the existing literature on beekeeping and climate change (see Section “Methods Used in the Text Corpus”). Most empirical studies rely on interviews, questionnaires, and surveys to elicit beekeepers’ perceptions, experiences, and self-reported responses to climatic change. While these approaches generate valuable insights, they are limited in their capacity to capture social practices as they are actually performed and should thus be complemented by methods that attend to what practitioners do, not only to what they say.

Beekeeping is a highly embodied, material, and temporally structured practice. As practitioners, beekeepers know more than they can tell, and much of what they know is tacit (cf. Meisch et al. 2022). Interviews and surveys therefore tend to privilege explicit reasoning over practical know-how and risk overlooking how competences, materials, meanings, timings, and more-than-human relations come together in everyday action.

Several studies in the text corpus already point beyond these limitations by employing focus groups, participant observation, or mixed-method approaches. Observational methods, in particular, make it possible to document how beekeepers interact with hives, tools, landscapes, and bees, how they attune to rhythms of weather and flowering, and how adaptive practices are improvised over time. Observation was used, for instance, by Mtengeti et al. (2013, p. 363) to collect ‘symbolic languages and responses from the study subjects as well as relevant practices’.

We suggest an expansion of the interdisciplinary methodological repertoire in bee research and a more vivid engagement with qualitative social science research (cf. also Lorenz 2021; Shanahan 2022). Although ethnographic approaches, long-term observation, or participatory methods are well established in other areas of beekeeping research (cf., e.g. Phillips 2014, 2020; Cilia 2019, 2020; Brown et al. 2024), their application in the context of climate change remains limited. A practice-based approach to bee research would further allow analysis beyond the focus on climate change adopted in this paper, addressing additional challenges currently affecting the social practice of beekeeping (cf., e.g. López-i-Gelats et al., 2025).

## Conclusion

Through the first systematic review to examine beekeeping explicitly as a social practice under climate change, we synthesise 27 peer-reviewed studies from diverse world regions and disciplines. Our analysis reveals that existing research has predominantly approached beekeeping through ecological and technical lenses, while it largely neglects *beekeeping as a social practice*. We identify this as a significant gap. Our analysis does not diminish the importance of these lenses but shows that they are only effective once they are embedded in broader practice reconfigurations of competences, materials, meanings, temporalities, and more-than-human relations.

Our research is relevant for an international readership as beekeeping is a globally distributed practice closely tied to biodiversity, agriculture, and rural livelihoods. By treating it as a climate-sensitive practice, we bring ecological and social sciences into closer dialogue and illuminate how socio-environmental linkages are enacted in everyday life. Social practice theory offers a promising interdisciplinary framework for understanding climate adaptation, with perspectives that are transferable to other fields of environmental research.

We suggest social practices—rather than individual behaviour—are a crucial social unit for studying and understanding how social fields persist and/or change (Bruns et al. 2022; Bremer et al. 2024). Social practices link the social, material and more-than-human worlds. Climatic change challenges the elements of social practices and the linkages between them alike. Adaptation, then, is the realignment of these elements. Studying social practices also brings into sight the many informal micro-adaptative practices within communities of practice, alongside formalised collective decision-making, and policy responses, thus providing insights into climate change adaptation on the ground. While not a social practice study itself, this advanced review was organised by social practice theory, and we assert that this can be a promising format for reviewing climate impacts on other fields of human (and more-than-human) activity.

We recommend, first, that climate adaptation be understood and supported as timely micro-adaptation within local social practices, recognising how practitioners reconfigure their activities in response to environmental change; and second, that beekeeping—like agricultural practices more broadly—be analysed through the lens of social practice, a perspective that calls for stronger inter- and transdisciplinary collaboration.
